# (*Z*)-3-(9-Anthr­yl)-1-(4-methoxy­phen­yl)prop-2-en-1-one^1^

**DOI:** 10.1107/S1600536809038665

**Published:** 2009-10-10

**Authors:** Suchada Chantrapromma, Jirapa Horkaew, Thitipone Suwunwong, Hoong-Kun Fun

**Affiliations:** a Crystal Materials Research Unit, Department of Chemistry, Faculty of Science, Prince of Songkla University, Hat-Yai, Songkhla 90112, Thailand; b X-ray Crystallography Unit, School of Physics, Universiti Sains Malaysia, 11800 USM, Penang, Malaysia

## Abstract

The title chalcone derivative, C_24_H_18_O_2_, which consists of the substituted 4-methoxy­phenyl and anthracene rings bridged by the prop-2-en-1-one unit, exists in a *cis* configuration. The mol­ecule is twisted, the inter­planar angle between the benzene and anthracene rings being 69.50 (10)°. The meth­oxy group is coplanar with the attached benzene ring [C—O—C—C angle = 2.9 (3)°]. In the crystal structure, mol­ecules are linked into chains along the *a* axis by a weak C—H⋯O(enone) inter­action. The chains are stacked along the *c* axis. A C—H⋯π inter­action involving the benzene ring is observed.

## Related literature

For bond-length data, see: Allen *et al.* (1987 ▶). For related structures, see: Fun *et al.* (2009 ▶); Suwunwong *et al.* (2009 ▶). For background to and applications of chalcones, see: Patil & Dharmaprakash (2008 ▶); Saydam *et al.* (2003 ▶); Svetlichny *et al.* (2007 ▶). For the stability of the temperature controller used in the data collection, see Cosier & Glazer, (1986 ▶).
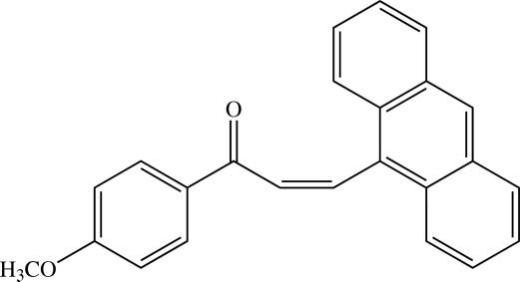


## Experimental

### 

#### Crystal data

C_24_H_18_O_2_
*M* *_r_* = 338.38
Monoclinic, 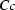

*a* = 5.5018 (2) Å
*b* = 19.9215 (8) Å
*c* = 16.0500 (7) Å
β = 95.072 (2)°
*V* = 1752.26 (12) Å^3^
*Z* = 4
Mo *K*α radiation
μ = 0.08 mm^−1^
*T* = 293 K
0.54 × 0.27 × 0.09 mm

#### Data collection

Bruker SMART APEXII CCD area-detector diffractometer
Absorption correction: multi-scan (*SADABS*; Bruker, 2005 ▶) *T*_min_ = 0.958, *T*_max_ = 0.993
7966 measured reflections
1721 independent reflections
1545 reflections with *I* > 2σ(*I*)
*R*_int_ = 0.023

#### Refinement

*R*[*F* ^2^ > 2σ(*F* ^2^)] = 0.037
*wR*(*F* ^2^) = 0.087
*S* = 1.07
1721 reflections
236 parameters
2 restraints
H-atom parameters constrained
Δρ_max_ = 0.14 e Å^−3^
Δρ_min_ = −0.14 e Å^−3^

### 

Data collection: *APEX2* (Bruker, 2005 ▶); cell refinement: *SAINT* (Bruker, 2005 ▶); data reduction: *SAINT*; program(s) used to solve structure: *SHELXTL* (Sheldrick, 2008 ▶); program(s) used to refine structure: *SHELXTL*; molecular graphics: *SHELXTL*; software used to prepare material for publication: *SHELXTL* and *PLATON* (Spek, 2009 ▶).

## Supplementary Material

Crystal structure: contains datablocks global, I. DOI: 10.1107/S1600536809038665/sj2659sup1.cif

Structure factors: contains datablocks I. DOI: 10.1107/S1600536809038665/sj2659Isup2.hkl

Additional supplementary materials:  crystallographic information; 3D view; checkCIF report

## Figures and Tables

**Table 1:** Hydrogen-bond geometry (Å, °)

*D*—H⋯*A*	*D*—H	H⋯*A*	*D*⋯*A*	*D*—H⋯*A*
C8—H8*A*⋯O1^i^	0.93	2.47	3.290 (3)	147
C24—H24*A*⋯O1^ii^	0.96	2.59	3.176 (4)	120
C17—H17*A*⋯*Cg*1^iii^	0.93	2.89	3.694 (3)	145

Symmetry codes: (i) 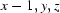; (ii) 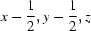; (iii) 
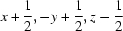
. *Cg*1 is the centroid of the C1–C6 ring.

**Fig. 1.**
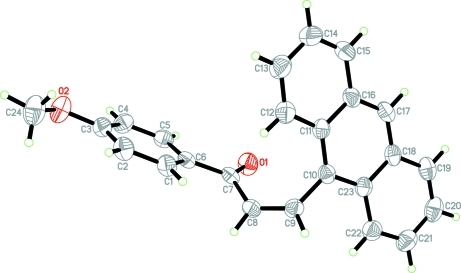
The molecular structure of the title compound, showing 50% probability displacement ellipsoids and the atom-numbering scheme.

**Fig. 2.**
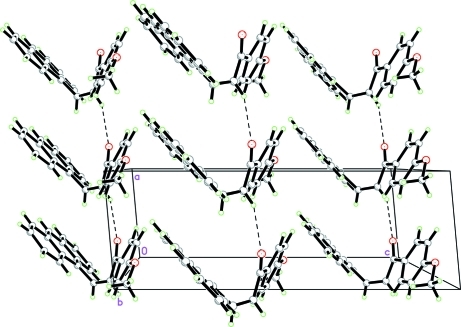
The crystal packing of the title compound viewed along the b axis, showing chains running along the a axis. Weak C—H···O interactions are shown as dashed lines.

## supplementary crystallographic information

### Comment

Chalcones have been studied for their wide range of applications such as non-linear optical (Patil & Dharmaprakash, 2008) and fluorescent properties (Svetlichny *et al.*, 2007) and biological activities (Saydam *et al.*, 2003). We have previously reported crystal structures of chalcone derivatives containing the anthracene moiety which exist in both the  *E* (Suwunwong *et al.*, 2009) and *Z* configurations (Fun *et al.*, 2009). The title compound was synthesized to study its fluorescent properties in addition to its antibacterial activity. The title compound shows interesting fluorescence properties which will be reported elsewhere. The crystal structure of the title compound was studied in order to elucidate its conformation which may affect the fluorescence properties.

The molecule of the title chalcone derivative, C_24_H_18_O_2_, (Fig. 1) exists in a *Z* configuration with respect to the C8=C9 ethenyl bond with the torsion angle C7–C8–C9–C10 being 3.6 (5)°. The anthracene ring system (C10–C23) is essentially planar with the root mean deviation of 0.050 (3) Å. The molecule is twisted as shown by the interplanar angle between the 4-methoxyphenyl and anthracene rings being 69.50 (10)°. The substituted methoxy group is coplanar with the phenyl ring with the torsion angle C24–O2–C3–C2 being 2.9 (3)°. The prop-2-en-1-one unit (C7—C9/O1) is twisted with the torsion angle O1–C7–C8–C9 of 44.5 (4)°. The orientation of the prop-2-en-1-one unit with respect to the 4-methoxyphenyl and anthracene rings is indicated by the torsion angles C1–C6–C7–C8 = 15.6 (4) and C7–C8–C9–C10 = 3.6 (5) °. The bond distances (Allen *et al.*, 1987) and angles are  normal and comparable to those found in closely related structures (Fun *et al.*, 2009; Suwunwong *et al.*, 2009).

In the crystal packing, the molecules are linked into chains along the *a* axis through the enone unit by a weak C8—H8A···O1 interaction (Fig. 2, Table 1). These chains are stacked along the *c* axis involving a C—H···π interaction (Table 1); *Cg*_1_ is the centroid of the C1–C6 ring.

### Experimental

The title compound was synthesized by condensation of anthracene-9-carbaldehyde (0.41 g, 2 mmol) with 4-methoxyacetophenone (0.30 g, 2 mmol) in ethanol (30 ml) in the presence of 30% aqueous NaOH (5 ml) at room temperature. After stirring for 3 hr, a yellow solid appeared and was then collected by filtration, washed with acetone and dried in air. Yellow block-shaped single crystals of the title compound suitable for *x*-ray structure determination were recrystalized from ethanol by the slow evaporation of the solvent at room temperature after several days, Mp. 440–441 K.

### Refinement

All H atoms were placed in calculated positions, with C—H = 0.93 Å, *U*_iso_ = 1.2*U*_eq_(C) for aromatic and CH and C—H = 0.96 Å, *U*_iso_ = 1.5*U*_eq_(C) for CH_3_ atoms. A rotating group model was used for the methyl groups. The highest residual electron density peak is located at 0.73 Å from C18 and the deepest hole is located at 1.39 Å from C17. A total of 1128 Friedel pairs were merged before final refinement as there is no large anomalous dispersion for the determination of the absolute structure.

### Crystal data

**Table:**

C_24_H_18_O_2_	*F*(000) = 712
*M**_r_* = 338.38	*D*_x_ = 1.283 Mg m^−^^3^
Monoclinic, *C**c*	Melting point = 440–441 K
Hall symbol: C -2yc	Mo *K*α radiation, λ = 0.71073 Å
*a* = 5.5018 (2) Å	Cell parameters from 1721 reflections
*b* = 19.9215 (8) Å	θ = 2.0–26.0°
*c* = 16.0500 (7) Å	µ = 0.08 mm^−^^1^
β = 95.072 (2)°	*T* = 293 K
*V* = 1752.26 (12) Å^3^	Plate, yellow
*Z* = 4	0.54 × 0.27 × 0.09 mm

### Data collection

**Table:**

Bruker SMART APEXII CCD area-detector diffractometer	1721 independent reflections
Radiation source: fine-focus sealed tube	1545 reflections with *I* > 2σ(*I*)
graphite	*R*_int_ = 0.023
Detector resolution: 8.33 pixels mm^-1^	θ_max_ = 26.0°, θ_min_ = 2.0°
ω scans	*h* = −6→6
Absorption correction: multi-scan (*SADABS*; Bruker, 2005)	*k* = −24→23
*T*_min_ = 0.958, *T*_max_ = 0.993	*l* = −19→19
7966 measured reflections	

### Refinement

**Table:**

Refinement on *F*^2^	Primary atom site location: structure-invariant direct methods
Least-squares matrix: full	Secondary atom site location: difference Fourier map
*R*[*F*^2^ > 2σ(*F*^2^)] = 0.037	Hydrogen site location: inferred from neighbouring sites
*wR*(*F*^2^) = 0.087	H-atom parameters constrained
*S* = 1.07	*w* = 1/[σ^2^(*F*_o_^2^) + (0.0414*P*)^2^ + 0.4575*P*] where *P* = (*F*_o_^2^ + 2*F*_c_^2^)/3
1721 reflections	(Δ/σ)_max_ = 0.001
236 parameters	Δρ_max_ = 0.14 e Å^−^^3^
2 restraints	Δρ_min_ = −0.14 e Å^−^^3^

### Special details

**Table:**

Geometry. All e.s.d.'s (except the e.s.d. in the dihedral angle between two l.s. planes) are estimated using the full covariance matrix. The cell e.s.d.'s are taken into account individually in the estimation of e.s.d.'s in distances, angles and torsion angles; correlations between e.s.d.'s in cell parameters are only used when they are defined by crystal symmetry. An approximate (isotropic) treatment of cell e.s.d.'s is used for estimating e.s.d.'s involving l.s. planes.
Refinement. Refinement of *F*^2^ against ALL reflections. The weighted *R*-factor *wR* and goodness of fit *S* are based on *F*^2^, conventional *R*-factors *R* are based on *F*, with *F* set to zero for negative *F*^2^. The threshold expression of *F*^2^ > σ(*F*^2^) is used only for calculating *R*-factors(gt) *etc*. and is not relevant to the choice of reflections for refinement. *R*-factors based on *F*^2^ are statistically about twice as large as those based on *F*, and *R*- factors based on ALL data will be even larger.

### Fractional atomic coordinates and isotropic or equivalent isotropic displacement parameters (Å^2^)

**Table:**

	*x*	*y*	*z*	*U*_iso_*/*U*_eq_	
O1	0.7379 (3)	0.26049 (10)	0.44324 (14)	0.0584 (5)	
O2	0.5958 (4)	−0.04663 (10)	0.52663 (15)	0.0663 (6)	
C1	0.3633 (5)	0.11209 (14)	0.43620 (17)	0.0469 (7)	
H1A	0.2261	0.1294	0.4055	0.056*	
C2	0.3691 (5)	0.04454 (15)	0.45676 (18)	0.0514 (7)	
H2A	0.2382	0.0169	0.4394	0.062*	
C3	0.5709 (5)	0.01859 (14)	0.50326 (17)	0.0469 (6)	
C4	0.7641 (5)	0.06087 (14)	0.52870 (18)	0.0498 (7)	
H4A	0.8992	0.0437	0.5607	0.060*	
C5	0.7586 (4)	0.12742 (14)	0.50729 (17)	0.0448 (6)	
H5A	0.8907	0.1548	0.5243	0.054*	
C6	0.5565 (4)	0.15470 (13)	0.46010 (15)	0.0394 (6)	
C7	0.5525 (4)	0.22645 (13)	0.43565 (16)	0.0413 (6)	
C8	0.3147 (4)	0.25664 (13)	0.40406 (18)	0.0454 (6)	
H8A	0.1795	0.2456	0.4322	0.054*	
C9	0.2783 (5)	0.29786 (13)	0.33936 (17)	0.0451 (6)	
H9A	0.1214	0.3150	0.3286	0.054*	
C10	0.4614 (4)	0.31959 (13)	0.28220 (16)	0.0411 (6)	
C11	0.5862 (5)	0.27224 (13)	0.23652 (16)	0.0428 (6)	
C12	0.5348 (6)	0.20192 (15)	0.23660 (18)	0.0527 (7)	
H12A	0.4126	0.1859	0.2678	0.063*	
C13	0.6601 (6)	0.15829 (16)	0.1923 (2)	0.0634 (9)	
H13A	0.6205	0.1129	0.1927	0.076*	
C14	0.8495 (7)	0.18016 (18)	0.1455 (2)	0.0670 (9)	
H14A	0.9371	0.1492	0.1167	0.080*	
C15	0.9040 (6)	0.24607 (17)	0.14224 (18)	0.0580 (8)	
H15A	1.0296	0.2601	0.1112	0.070*	
C16	0.7721 (5)	0.29480 (14)	0.18577 (15)	0.0460 (6)	
C17	0.8194 (5)	0.36309 (15)	0.17973 (16)	0.0491 (7)	
H17A	0.9427	0.3773	0.1478	0.059*	
C18	0.6874 (5)	0.41081 (14)	0.22015 (16)	0.0463 (7)	
C19	0.7276 (6)	0.48094 (15)	0.21182 (19)	0.0559 (8)	
H19A	0.8496	0.4957	0.1796	0.067*	
C20	0.5933 (6)	0.52656 (16)	0.2495 (2)	0.0622 (8)	
H20A	0.6211	0.5721	0.2422	0.075*	
C21	0.4106 (6)	0.50528 (17)	0.2999 (2)	0.0616 (8)	
H21A	0.3169	0.5370	0.3251	0.074*	
C22	0.3704 (5)	0.43918 (16)	0.31209 (18)	0.0529 (7)	
H22A	0.2533	0.4263	0.3473	0.063*	
C23	0.5028 (5)	0.38878 (13)	0.27229 (16)	0.0426 (6)	
C24	0.4045 (7)	−0.09228 (16)	0.4985 (3)	0.0761 (10)	
H24A	0.4477	−0.1368	0.5173	0.114*	
H24B	0.3820	−0.0916	0.4385	0.114*	
H24C	0.2557	−0.0790	0.5209	0.114*	

### Atomic displacement parameters (Å^2^)

**Table:**

	*U*^11^	*U*^22^	*U*^33^	*U*^12^	*U*^13^	*U*^23^
O1	0.0426 (10)	0.0571 (12)	0.0750 (14)	−0.0105 (10)	0.0021 (9)	0.0098 (11)
O2	0.0777 (15)	0.0442 (12)	0.0741 (15)	−0.0021 (11)	−0.0085 (12)	0.0055 (11)
C1	0.0362 (13)	0.0538 (17)	0.0499 (15)	−0.0038 (12)	−0.0007 (11)	0.0081 (13)
C2	0.0466 (15)	0.0532 (17)	0.0532 (16)	−0.0111 (13)	−0.0017 (13)	0.0015 (13)
C3	0.0513 (15)	0.0464 (16)	0.0435 (15)	0.0022 (12)	0.0071 (12)	0.0015 (12)
C4	0.0450 (15)	0.0519 (17)	0.0514 (16)	0.0072 (13)	−0.0026 (12)	0.0011 (13)
C5	0.0340 (13)	0.0535 (16)	0.0471 (15)	−0.0037 (12)	0.0041 (11)	−0.0032 (12)
C6	0.0337 (12)	0.0493 (16)	0.0365 (13)	−0.0027 (11)	0.0101 (10)	0.0015 (11)
C7	0.0372 (13)	0.0498 (15)	0.0387 (13)	−0.0049 (12)	0.0132 (11)	0.0020 (12)
C8	0.0357 (13)	0.0479 (15)	0.0543 (16)	−0.0020 (12)	0.0133 (11)	0.0064 (13)
C9	0.0363 (13)	0.0449 (16)	0.0542 (15)	0.0019 (11)	0.0042 (11)	0.0035 (13)
C10	0.0381 (13)	0.0470 (16)	0.0377 (13)	−0.0006 (11)	−0.0003 (10)	0.0045 (11)
C11	0.0459 (14)	0.0439 (15)	0.0376 (13)	0.0007 (11)	−0.0018 (11)	0.0044 (12)
C12	0.0589 (17)	0.0486 (16)	0.0505 (16)	−0.0023 (14)	0.0040 (14)	0.0033 (14)
C13	0.084 (2)	0.0470 (17)	0.0592 (18)	0.0052 (16)	0.0048 (17)	−0.0025 (15)
C14	0.084 (2)	0.063 (2)	0.0557 (18)	0.0208 (18)	0.0146 (17)	−0.0049 (16)
C15	0.0646 (19)	0.068 (2)	0.0430 (16)	0.0071 (16)	0.0165 (14)	0.0037 (14)
C16	0.0495 (16)	0.0547 (17)	0.0335 (13)	0.0020 (13)	0.0015 (11)	0.0041 (12)
C17	0.0483 (15)	0.0615 (19)	0.0384 (14)	−0.0058 (13)	0.0083 (12)	0.0093 (13)
C18	0.0499 (15)	0.0505 (17)	0.0370 (13)	−0.0056 (13)	−0.0043 (12)	0.0073 (12)
C19	0.0635 (19)	0.0537 (19)	0.0496 (16)	−0.0158 (15)	0.0000 (14)	0.0084 (14)
C20	0.079 (2)	0.0415 (17)	0.063 (2)	−0.0042 (16)	−0.0104 (17)	0.0035 (15)
C21	0.070 (2)	0.0494 (19)	0.0640 (19)	0.0076 (15)	−0.0005 (16)	−0.0029 (15)
C22	0.0531 (16)	0.0531 (19)	0.0520 (17)	0.0021 (14)	0.0027 (13)	0.0015 (14)
C23	0.0438 (14)	0.0436 (15)	0.0390 (13)	−0.0005 (12)	−0.0037 (11)	0.0029 (11)
C24	0.093 (3)	0.052 (2)	0.082 (2)	−0.0170 (18)	−0.003 (2)	0.0025 (18)

### Geometric parameters (Å, °)

**Table:**

O1—C7	1.222 (3)	C12—H12A	0.9300
O2—C3	1.356 (3)	C13—C14	1.407 (5)
O2—C24	1.433 (4)	C13—H13A	0.9300
C1—C2	1.385 (4)	C14—C15	1.349 (5)
C1—C6	1.387 (4)	C14—H14A	0.9300
C1—H1A	0.9300	C15—C16	1.431 (4)
C2—C3	1.382 (4)	C15—H15A	0.9300
C2—H2A	0.9300	C16—C17	1.390 (4)
C3—C4	1.389 (4)	C17—C18	1.391 (4)
C4—C5	1.369 (4)	C17—H17A	0.9300
C4—H4A	0.9300	C18—C19	1.423 (4)
C5—C6	1.399 (4)	C18—C23	1.440 (4)
C5—H5A	0.9300	C19—C20	1.348 (5)
C6—C7	1.482 (4)	C19—H19A	0.9300
C7—C8	1.488 (4)	C20—C21	1.411 (5)
C8—C9	1.325 (4)	C20—H20A	0.9300
C8—H8A	0.9300	C21—C22	1.352 (5)
C9—C10	1.486 (4)	C21—H21A	0.9300
C9—H9A	0.9300	C22—C23	1.425 (4)
C10—C23	1.408 (4)	C22—H22A	0.9300
C10—C11	1.410 (4)	C24—H24A	0.9600
C11—C12	1.429 (4)	C24—H24B	0.9600
C11—C16	1.435 (4)	C24—H24C	0.9600
C12—C13	1.350 (4)		
			
C3—O2—C24	117.9 (2)	C12—C13—H13A	119.4
C2—C1—C6	121.8 (3)	C14—C13—H13A	119.4
C2—C1—H1A	119.1	C15—C14—C13	120.0 (3)
C6—C1—H1A	119.1	C15—C14—H14A	120.0
C3—C2—C1	119.5 (3)	C13—C14—H14A	120.0
C3—C2—H2A	120.2	C14—C15—C16	121.2 (3)
C1—C2—H2A	120.2	C14—C15—H15A	119.4
O2—C3—C2	124.4 (3)	C16—C15—H15A	119.4
O2—C3—C4	116.4 (2)	C17—C16—C15	121.6 (3)
C2—C3—C4	119.3 (3)	C17—C16—C11	119.5 (2)
C5—C4—C3	120.9 (2)	C15—C16—C11	118.9 (3)
C5—C4—H4A	119.5	C16—C17—C18	121.9 (2)
C3—C4—H4A	119.5	C16—C17—H17A	119.1
C4—C5—C6	120.7 (2)	C18—C17—H17A	119.1
C4—C5—H5A	119.6	C17—C18—C19	122.4 (3)
C6—C5—H5A	119.6	C17—C18—C23	119.1 (2)
C1—C6—C5	117.7 (2)	C19—C18—C23	118.5 (3)
C1—C6—C7	121.6 (2)	C20—C19—C18	121.6 (3)
C5—C6—C7	120.7 (2)	C20—C19—H19A	119.2
O1—C7—C6	121.1 (2)	C18—C19—H19A	119.2
O1—C7—C8	120.7 (2)	C19—C20—C21	120.1 (3)
C6—C7—C8	118.2 (2)	C19—C20—H20A	119.9
C9—C8—C7	125.8 (2)	C21—C20—H20A	119.9
C9—C8—H8A	117.1	C22—C21—C20	120.6 (3)
C7—C8—H8A	117.1	C22—C21—H21A	119.7
C8—C9—C10	126.9 (2)	C20—C21—H21A	119.7
C8—C9—H9A	116.5	C21—C22—C23	121.7 (3)
C10—C9—H9A	116.5	C21—C22—H22A	119.2
C23—C10—C11	120.3 (2)	C23—C22—H22A	119.2
C23—C10—C9	118.7 (2)	C10—C23—C22	123.0 (2)
C11—C10—C9	120.9 (2)	C10—C23—C18	119.6 (2)
C10—C11—C12	123.4 (2)	C22—C23—C18	117.4 (2)
C10—C11—C16	119.3 (2)	O2—C24—H24A	109.5
C12—C11—C16	117.3 (2)	O2—C24—H24B	109.5
C13—C12—C11	121.4 (3)	H24A—C24—H24B	109.5
C13—C12—H12A	119.3	O2—C24—H24C	109.5
C11—C12—H12A	119.3	H24A—C24—H24C	109.5
C12—C13—C14	121.2 (3)	H24B—C24—H24C	109.5
			
C6—C1—C2—C3	0.8 (4)	C12—C13—C14—C15	−2.0 (5)
C24—O2—C3—C2	2.6 (4)	C13—C14—C15—C16	0.0 (5)
C24—O2—C3—C4	−177.4 (3)	C14—C15—C16—C17	−177.0 (3)
C1—C2—C3—O2	−179.8 (3)	C14—C15—C16—C11	2.8 (4)
C1—C2—C3—C4	0.1 (4)	C10—C11—C16—C17	−2.6 (4)
O2—C3—C4—C5	179.0 (3)	C12—C11—C16—C17	176.3 (2)
C2—C3—C4—C5	−1.0 (4)	C10—C11—C16—C15	177.6 (2)
C3—C4—C5—C6	0.8 (4)	C12—C11—C16—C15	−3.4 (3)
C2—C1—C6—C5	−1.0 (4)	C15—C16—C17—C18	178.3 (2)
C2—C1—C6—C7	178.0 (2)	C11—C16—C17—C18	−1.5 (4)
C4—C5—C6—C1	0.1 (4)	C16—C17—C18—C19	−177.5 (3)
C4—C5—C6—C7	−178.9 (2)	C16—C17—C18—C23	2.4 (4)
C1—C6—C7—O1	−166.3 (3)	C17—C18—C19—C20	178.0 (3)
C5—C6—C7—O1	12.6 (4)	C23—C18—C19—C20	−1.8 (4)
C1—C6—C7—C8	15.6 (4)	C18—C19—C20—C21	1.2 (5)
C5—C6—C7—C8	−165.4 (2)	C19—C20—C21—C22	0.9 (5)
O1—C7—C8—C9	44.5 (4)	C20—C21—C22—C23	−2.5 (5)
C6—C7—C8—C9	−137.5 (3)	C11—C10—C23—C22	175.5 (2)
C7—C8—C9—C10	3.6 (5)	C9—C10—C23—C22	−2.5 (4)
C8—C9—C10—C23	−124.0 (3)	C11—C10—C23—C18	−4.8 (3)
C8—C9—C10—C11	58.1 (4)	C9—C10—C23—C18	177.2 (2)
C23—C10—C11—C12	−173.1 (2)	C21—C22—C23—C10	−178.5 (3)
C9—C10—C11—C12	4.8 (4)	C21—C22—C23—C18	1.8 (4)
C23—C10—C11—C16	5.7 (4)	C17—C18—C23—C10	0.8 (3)
C9—C10—C11—C16	−176.3 (2)	C19—C18—C23—C10	−179.3 (2)
C10—C11—C12—C13	−179.6 (3)	C17—C18—C23—C22	−179.5 (2)
C16—C11—C12—C13	1.5 (4)	C19—C18—C23—C22	0.4 (3)
C11—C12—C13—C14	1.2 (5)		

### Hydrogen-bond geometry (Å, °)

**Table:**

*D*—H···*A*	*D*—H	H···*A*	*D*···*A*	*D*—H···*A*
C8—H8A···O1^i^	0.93	2.47	3.290 (3)	147
C24—H24A···O1^ii^	0.96	2.59	3.176 (4)	120
C17—H17A···Cg1^iii^	0.93	2.89	3.694 (3)	145

Symmetry codes: (i) *x*−1, *y*, *z*; (ii) *x*−1/2, *y*−1/2, *z*; (iii) *x*+1/2, −*y*+1/2, *z*−1/2.
